# A Cold-Shock Protein from the South Pole-Dwelling Soil Bacterium *Arthrobacter* sp. Confers Cold Tolerance to Rice

**DOI:** 10.3390/genes12101589

**Published:** 2021-10-09

**Authors:** So Young Kim, Joung Sug Kim, Woosuk Cho, Kyong Mi Jun, Xiaoxuan Du, Kyung Do Kim, Yeon-Ki Kim, Gang-Seob Lee

**Affiliations:** 1 Biosafety Division, National Institute of Agricultural Sciences, Jeonju 54874, Korea; hotmilk73@hotmail.com (S.Y.K.); phyto@korea.kr (W.C.); haobingshuaike@hotmail.com (X.D.); 2 Department of Biosciences and Bioinformatics, Myongji University, 116 Myongji-ro, Cheoin-gu, Yongin 17060, Korea; llmon7711@gmail.com (J.S.K.); kyungdokim@mju.ac.kr (K.D.K.); 3 Genomics Genetics Institute, GreenGene BioTech, Inc., 16-4 Dongbaek jungang-ro 16beon-gil, Giheung-gu, Yongin 17015, Korea; queenmi76@gmail.com

**Keywords:** cold-shock protein, *Arthrobacter*, rice, low temperature, RNA sequencing

## Abstract

Low temperature is a critical environmental factor restricting the physiology of organisms across kingdoms. In prokaryotes, cold shock induces the expression of various genes and proteins involved in cellular processes. Here, a cold-shock protein (ArCspA) from the South Pole-dwelling soil bacterium *Arthrobacter* sp. A2-5 was introduced into rice, a monocot model plant species. Four-week-old *35S:ArCspA* transgenic rice plants grown in a cold chamber at 4 °C survived for 6 days. Cold stress significantly decreased the chlorophyll content in WT plants after 4 days compared with that in *35S:ArCspA* transgenic plants. RNA-seq analysis was performed on WT and *35S:ArCspA* transgenic rice with/without cold stress. GO terms such as “response to stress (GO:0006950)”, “response to cold (GO:0009409)”, and “response to heat (GO:0009408)” were significantly enriched among the upregulated genes in the *35S:ArCspA* transgenic rice under normal conditions, even without cold-stress treatment. The expression of five cold stress-related genes, *Rab16B* (Os11g0454200), *Rab21* (Os11g0454300), *LEA22* (Os01g0702500), *ABI5* (Os01 g0859300), and *MAPK5* (Os03g0285800), was significantly upregulated in the transgenic rice compared with the WT rice. These results indicate that the *ArCspA* gene might be involved in the induction of cold-responsive genes and provide cold tolerance.

## 1. Introduction

Organisms have evolved elaborate mechanisms to cope with abiotic stresses such as dehydration, heat shock and low-temperature stress. Low temperature is a critical environmental factor that restricts the growth and development of many plant species, including rice [1]. Given that rice evolved under a subtropical climate but then expanded into temperate zones through domestication by humans, it may be susceptible to cold stress [2]. Plant tolerance to low temperature can be classified into two types: chilling tolerance (above 0 °C) and freezing tolerance (below 0 °C). Under chilling-stress conditions, stomatal conductance, photosynthesis, growth and, hence, productivity are hindered due to the solidification of membrane lipids and, consequently, the deactivation of carrier-mediated transport and enzyme-mediated processes [3]. Often, reactive oxygen species (ROS) are generated due to the excess excitation energy in the electron transport chain of photosynthesis. If plants are exposed to freezing conditions, the damage is even more severe because osmotic stress-induced dehydration causes ice crystals to form in the cell walls. Under these conditions, plants are also exposed to stresses that are very similar to stresses caused by water deficit and mechanical processes.

Cold acclimation is a process in which temperate plants acquire freezing tolerance upon previous exposure to low but non-freezing temperatures, and the survival rate increases under subsequent freezing conditions [4,5]. During the cold acclimation process, the lipid composition and proteins in the plasma membrane dynamically respond, as has been shown in various plants, and the antisense suppression of phospholipase Dα1 (PLDα1) increases the freezing tolerance [6]. In this process, compatible solutes such as simple sugars (sucrose, glucose, fructose, and raffinose), proline, and glycine betaine accumulate at high concentrations in the cytoplasm. These factors contribute to balancing external osmotic pressure by allowing plants to lower their water potential and maintain turgor pressure without a disruption in cellular activity.

Plant cells have evolved dedicated systems that respond to cold stress, transduce signals, and induce numerous genes that function in the cold-stress response and cold tolerance. Increased [Ca2+]cyt through mechanosensitive mechanisms or signal transduction might be among the immediate responses. The mechanosensitive Ca2+-permeable channels Mid1-Complementing Activity 1 (MCA1) and MCA2 may contribute to the influx of Ca2+ [7]. CHILLING TOLERANCE DIVERGENCE 1 (COLD1), a regulator of G protein signaling that localizes to the plasma membrane, interacts with G protein α-subunits to activate Ca2+ channels and subsequent Ca2+ flux [8]. Annexins compose a subfamily of phospholipid and Ca2+-binding proteins that mediate the generation of cold-induced Ca2+ signals [9,10]. Following signal transduction, numerous genes are induced; interestingly, many of the cold-regulated genes contain C-repeat (CRT) elements or dehydration-responsive elements (DREs) in their promoter regions that bind to CBF/DREB1 transcription factors belonging to the APETALA2/ethylene-responsive factor (ERF) superfamily [5,11,12,13,14]. CBF proteins bind directly to the promoters of cold-regulated (COR) genes, some of which encode cryoprotective proteins or are involved in the accumulation of osmolytes that enhance plant freezing tolerance [15,16]. In addition, many cold-responsive genes have been shown to be involved in dehydration and abscisic acid (ABA) responsiveness. The promoter regions of these genes also contain ABREs that might interact with pathway-specific transcription factors.

Low-temperature stress of prokaryotes such as *Escherichia coli* has been studied via abrupt shifts in the temperature of culture media—from 37–40 °C to 7–10 °C—which imposes stress on different proteins involved in various cellular processes, such as transcription, translation, mRNA degradation, and DNA recombination [17,18]. Among these proteins, cold-shock proteins (Csps) also accumulate [19]. In the *E. coli* genome, nine members of the *Csp* gene family (*cspA* to *cspI*) have been identified, and four have been found to be induced in response to cold shock [20]. All Csp family members possess the RNA-binding motifs RNP-1 (KGFGFI) and RNP-2 (VFVHF) [21]. *CspA*, the major *Csp* of *E. coli*, accumulates up to 10% of total proteins during cold stress and functions as an RNA chaperone to destabilize RNA secondary structure, enabling efficient translation at low temperatures [19]. In addition, *CspA*, *CspC*, and *CspE* regulate the expression of a set of cold-inducible genes [22]. *Csps* are widely conserved from bacteria to higher plants and animals. The overexpression of *SeCspA* and *SeCspB*, which were modified to plant-preferred codons from *E. coli*, appeared to increase salt, cold, and drought tolerance in transgenic Arabidopsis plants [23]. In Arabidopsis, *AtCsp3* knockout mutants and *AtCsp3* overexpression plants (At2g17870) exhibit freezing-sensitive and freezing-tolerant phenotypes, respectively [24]. *AtCsp2* plays an important role in plant development by negatively regulating flowering time and positively regulating seed/embryo development [25]. Csps commonly display an RNA chaperone activity and function as regulatory proteins in plants. In addition, wheat *Csp* (*WCsp1*) complements the cold-sensitive phenotype of the *E. coli csp* mutant [26]. *E. coli CspA* and *CspB* provide cold tolerance when overexpressed in Arabidopsis [27]. The evolutionarily conserved structures and biochemical activities of Csps suggest that these proteins are essential for the cold adaptations of both prokaryotes and eukaryotes.

In a previous study, we isolated *CspA* from the South Pole-dwelling soil bacterium *Arthrobacter* sp. A2-5 (*ArCspA*); the sequence of *CspA* is strikingly similar to that of *E. coli* CspA cold-shock protein family members and contains 2 RNA-binding motifs, RNP-1 (KGFGFI) and RNP-2 (VFVHF), which function as ssDNA chaperones [28]. We previously reported that the constitutive expression of *ArCspA* in tobacco plants resulted in cold-stress tolerance [28]. *ArCspA* might have undergone a subtly different evolutionary path from prokaryotes and dicotyledonous plants, so we analyzed the overexpression of *ArCspA* in rice, a monocot model plant species. The *ArCspA* gene improves the cold-stress tolerance of transgenic rice plants by modulating the expression levels of downstream cold stress-related genes.

## 2. Materials and Methods

### 2.1. Plant Materials and Growth Conditions

In a previous study, *ArCspA* (a cold-shock protein) was overexpressed under the control of the CaMV 35S promoter (*35S:ArCspA*), after which transgenic rice plants were generated through *Agrobacterium*-mediated transformation via phosphinothricin resistance selection on selective media [29]. Rice cultivar Dongjin (Oryza sativa ssp. Japonica cv. Dongjin) was used in this study. Among 118 independent *35S:ArCspA* transgenic rice plants (T0 generation), 52 lines were determined to have single copy numbers via TaqMan copy number assays [29,30]. Homozygous T3 lines were obtained and used for further experiments.

### 2.2. Determination of Relative Copy Numbers via TaqMan Real-Time Polymerase Chain Reaction (PCR)

To determine the copy numbers, genomic DNA was extracted from leaf samples of transgenic and wild-type (WT) plants. The rice *tubulin α-1* gene (AK102560) was used as an endogenous control, and its sequence is listed in Supplementary Table S1. For the transgene, we designed primers and a probe specific to the terminator of the *NOS* gene. FAM and VIC reporter dyes were attached to the 5′ end of the tub probe and NOS probe, respectively, while a nonfluorescent quencher (NFQ) and a minor groove binder (MGB) were attached to the 3′ end of these probes. Each PCR was carried out on an Applied Biosystems StepOnePlus Real-Time PCR System (Applied Biosystems, Waltham, MA, USA), with one cycle of 50 °C for 2 min and 95 °C for 10 min, followed by 40 cycles of 94 °C for 15 s and 60 °C for 60 s. Each reaction mixture consisted of 10 µL of TaqMan Master Mix (Applied Biosystems), 30 ng of genomic DNA, and an optimal concentration of each gene-specific primer and probe (each primer at 900 nM and each probe at 200 nM) in a total volume of 20 µL. To calculate the gene copy numbers, the relative quantitation analysis of the genomic DNA targets with real-time PCR data was performed using Applied Biosystems CopyCaller Software 2.0 (Applied Biosystems, USA) according to the manufacturer’s instructions.

### 2.3. RNA Extraction, cDNA Synthesis, and Quantitative Real-Time PCR Analysis

The total RNA was extracted from the leaves of WT and transgenic rice plants using an RNeasy Plant Mini Kit (Qiagen). Afterwards, 1 µg of total RNA was used as a template for reverse transcriptase (RT) reactions using a RevertAid H Minus First-Strand cDNA Synthesis Kit (Fermentas, USA), according to the manufacturer’s instructions. The cDNA products were diluted five-fold prior to use in quantitative real-time PCR analysis. Gene-specific primers were designed using the Primer-BLAST tool (http://ncbi.nlm.nih.gov) and are listed in Supplementary Table S1. As the endogenous control, the primers and probes were obtained from a predesigned TaqMan^®^ copy number assay of the rice *tubulin α*-*1 chain* gene (AK102560). PCR was performed on a 20-μL reaction mixture including 2 μL of rice cDNA, gene-specific primers at 0.1 µM, and 10 μL of 2× PCR master mix (SolGent, Republic of Korea). The reactions included an initial 10-min denaturation at 94 °C, followed by 40 cycles of PCR (95 °C for 30 s, 55 °C for 30 s, and 72 °C for 30 s) performed on a CFX96 real-time PCR detection system (Bio-Rad, Hercules, CA, USA), according to the manufacturers’ instructions. The expression level of OsActin1 was used for normalization of the real-time PCR results. Changes in the gene expression were analyzed using CFX Manager software (Bio-Rad, Hercules, CA, USA). Quantitative real-time PCR (RT-qPCR) was carried out for three technical repeats and three biological replicates.

### 2.4. Cold Treatment of Rice Plants

WT and *35S:ArCspA* transgenic rice plants were grown on soil for 4 weeks under 16-h light and 30 °C/8-h darkness, and at 20 °C cycle in a greenhouse. For cold-stress treatment, WT and transgenic rice plants were transferred to a cold chamber at 4 °C for 6 days and then allowed to recover for 2 days in a greenhouse. The degree of cold damage to the leaves was observed daily according to an indicator ranging from 1 (normal) to 5 (necrosis caused by very severe cold damage). After 13–27 days, the survival rate was calculated as the number of surviving plants. The experiments were repeated three times, each yielding similar results.

### 2.5. Chlorophyll Measurements

The chlorophyll contents were measured as described previously [31]. One hundred milligrams of leaves were harvested, mixed well together with dimethyl formamide (DMF), and were centrifuged at 13,000× *g* for 10 min at 4 °C. The chlorophyll contents were measured spectrophotometrically (663 nm for chlorophyll b and 645 nm for chlorophyll a).

Chlorophyll a = 12.7 × A663 nm – 2.79 × A645 nm

Chlorophyll b = 20.7 × A645 nm ‒ 4.62 × A663 nm

Total chlorophyll = 17.9 × A645 nm + 8.08 × A663 nm

(A—absorbance; pigment concentration in milligrams per gram of fresh weight (FW)).

### 2.6. RNA Sequencing (RNA-seq) and Data Processing

mRNA was purified using a TruSeq RNA sample preparation kit (Illumina, USA) following the manufacturer’s protocol. After purification, the mRNA was fragmented, and first-strand cDNA was generated using reverse transcriptase and random primers. Second-strand cDNA synthesis was then performed using DNA polymerase I and RNase H. These cDNA fragments were subsequently subjected to an end-repair process, polyadenylation, and adaptor ligation. To generate a cDNA library, the products were then purified and amplified via PCR in a bridged amplification reaction that occurred on the surface of the flow cell. A flow cell containing millions of unique clusters was then loaded into a NovaSeq 6000 device for automated cycles of extension and imaging. Each sequencing cycle was carried out in the presence of all four nucleotides, generating a series of images, each representing a single base extension at a specific cluster. During the initial sequencing, 3.7–5.0 Gb were sequenced at each end. These results generated 37–49 × 10^6^ paired-end reads (Table S2). For statistical analysis, two biological repeats for the control and line #2 without or with cold stress treatment was performed.

The raw sequence reads were mapped to the rice genome sequence (IRGSP-1.0_2014-06-25) in the Rice Annotation Project (RAP) database (http://rapdb.lab.nig.ac.jp). Reads in FASTQ files were aligned using HISAT2 based on HISAT and Bowtie2 implementations with the --dta-cufflinks option [32]. Within the program, SAMtools 1.2 was used for indexing and sorting BAM and SAM files. To extract the exons among the transcripts, we defined non-overlapping exonic regions with dexseq_prepare_annotation.py, and then extracted the per-exon read counts with the script dexseq_count.py using the annotated gtf file [33] in the DEXSeq package. The program returned one file for each biological replicate with the exon counts. We tested the differential exon usage with DEXseq and limma packages in the Bioconductor (bioconductor.org). The median count was shifted by adding 1 to avoid trivial division error.

## 3. Results

### 3.1. Expression of 35S:ArCspA Transgenic Rice

To test the effects of *ArCspA* in rice, plants were transformed with a vector harboring ArCspA under the control of the CaMV 35S promoter [29]. Among 118 independent transgenic rice plants, 52 lines were determined via TaqMan copy number assays to have single copy numbers [29]. Among them, by analyzing the flanking sequence tags (FSTs), we selected 11 transgenic lines whose transgenes were inserted into an intergenic region and designated them as *35S:ArCspA* lines [29,30].

**Figure 1:**
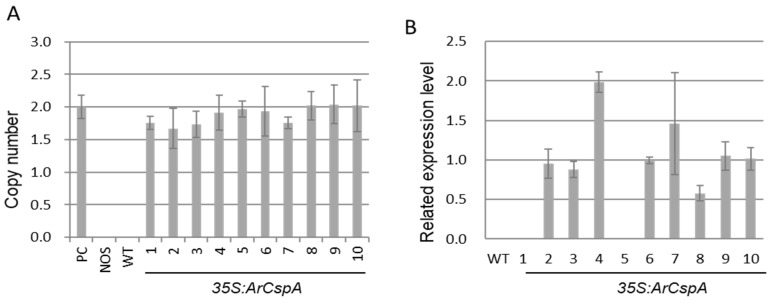
Copy numbers (**A**) and expression levels (**B**) of ArCspA genes in *35S:ArCspA* transgenic rice. (**A**) The copy numbers were determined via TaqMan real-time PCR. The *NOS* terminator and *tubulin-α-1* gene were used for T-DNA detection and as endogenous controls, respectively. The previously isolated single-copy homozygous transgenic line was used as a positive control (PC). (**B**) The relative expression levels of ArCspA were analyzed in *35S:ArCspA* transgenic plants via RT-qPCR analysis. The transcript levels were normalized to OsActin1 expression levels. The error bars indicate the ranges of the calculated values of three replicates for each sample.

First, the copy numbers and expression levels of ten transgenic rice plants were determined via RT-qPCR. To identify transgenic lines with a single copy of the T-DNA insertion, we performed TaqMan real-time PCR, which involved two probes: a *NOS* terminator for T-DNA detection and the *tubulin α-1* gene as an endogenous control (Figure 1A). The previously isolated single-copy homozygous transgenic line was used as a positive control (enrichment value of a single copy number = 2). When the enrichment value of T-DNA in each transgenic line was compared with that of the positive control, all the transgenic rice plants had one insertion copy number (Figure 1A). The transcript levels of *ArCspA* were significantly increased in the eight transgenic plants, excluding lines 1 and 5, which were not expressed like those in the WT plants (Figure 1B).

### 3.2. Effects of ArCspA Overexpression under Cold-Stress Conditions

**Figure 2:**
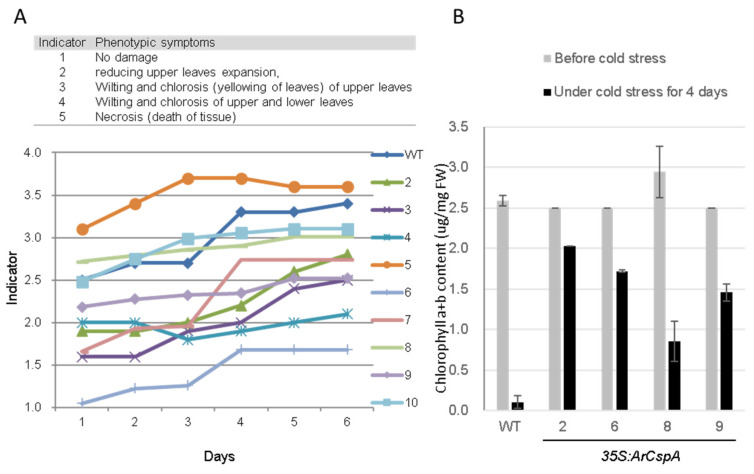
Performance of *35S:ArCspA* transgenic rice under cold stress. (**A**) Degree of cold damage in the WT, null (line #5), and eight independent transgenic lines for 6 days, with indicators ranging from 1 (normal) to 5 (necrosis from very severe cold damage). (**B**) Chlorophyll a+b content in *35S:ArCspA* transgenic rice (lines #2, 6, 8, and 9) before and after 4 days of cold stress.

To investigate whether the overexpression of *ArCspA* is correlated with tolerance to cold stress in transgenic plants, we assessed the effects of cold stress on *35S:ArCspA* transgenic rice plants. After they grew for 4 weeks, eight lines of *35S:ArCspA* transgenic rice plants, null plants (line #5), and WT plants were transferred to a cold chamber at 4 °C. We observed the degree of cold damage daily for 6 days, with an indicator ranging from 1 (normal) to 5 (necrosis from very severe cold damage) (Figure 2A). 

**Figure 3:**
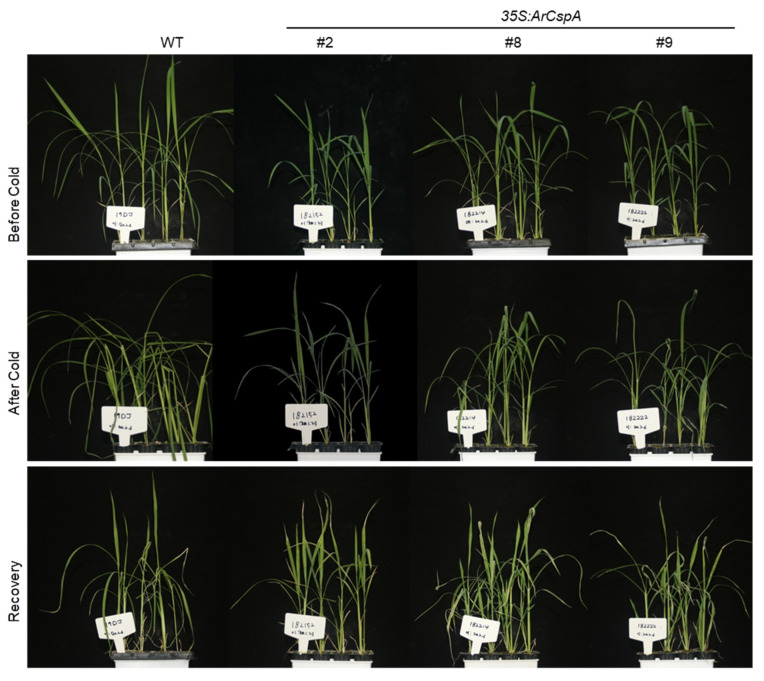
Comparison of the cold-stress tolerance of WT and *35S:ArCspA* transgenic rice. Four-week-old WT and *35S:ArCspA* (lines #2, 8, and 9) rice plants were subjected to cold treatment at 4 °C for 6 days, and then allowed to recover under normal conditions at 28 °C for 3 weeks.

The upper leaves of the WT plants and null plants began wilting after 1 and 3 days, respectively, but those of the *35S:ArCspA* transgenic plants wilted more slowly than the upper leaves of the WT plants (Figure 2A). Four transgenic plants (lines #2, 6, 8, and 9) were selected for measuring the chlorophyll content before and after 4 days of cold stress. Cold stress significantly decreased the chlorophyll content in the WT plants after 4 days compared with that in *35S:ArCspA* transgenic plants (Figure 2B). We then selected three transgenic plants (lines #2, 8, and 9) to observe their phenotype after the 3-week recovery period. The transgenic plants recovered much better than the WT plants did under normal temperature after cold stress (Figure 3). Taken together, these results showed that the constitutive expression of *ArCspA* increases the tolerance of rice to cold stress.

### 3.3. Transcriptome Analysis of 35S:ArCspA Transgenic Plants under Normal and Cold-Stress Conditions

Given that *ArCspA*-overexpressing rice plants are more tolerant to cold stress than WT plants, we next performed a transcriptomic analysis of *35S:ArCspA* transgenic rice and WT rice. To identify the downstream target genes of *ArCspA* responsible for improved cold tolerance, we conducted RNA-seq analysis using the total RNA from 4-week-old leaves of WT and *35S:ArCspA* transgenic rice plants grown under either normal (before) or cold-stress (after) conditions. Transgenic line #2 was selected for the RNA-seq analysis because it recovered better than the other lines. Two biological repeats were performed for the wild type and line #2. The raw reads were mapped to the rice reference genome (IRGSP-1.0). The expression values were measured as count values, and the DEGs were determined.

Among the 52,817 transcripts whose sequences were housed in the IRGSP RAP3 database (https://rapdb.dna.affrc.go.jp/), 60 to 70% of the transcripts were counted at least once (Table S3). For B (WT before cold stress), the mean count was 201.7 (s.d. of 1735.5), and the transcript with the most counts was Os09t0346500-04 (chlorophyll a-b binding protein), with 190,050 counts. However, for B2 (line #2 of *35S:ArCspA* before cold stress), the counts of Os09t0346500-04 was 169,854.3 which was 10 % lower than that of the WT (Table S3). For WT plants after cold stress, the mean count number was 325.8 (s.d. of 1868), and the transcript with the most counts was Os05t0129300-02 basic/leucine zipper protein, with 151,146.8 counts, the number of which decreased in A2 (line #2, of *35S:ArCspA* after cold stress) by 56% (Table S3).

**Figure 4:**
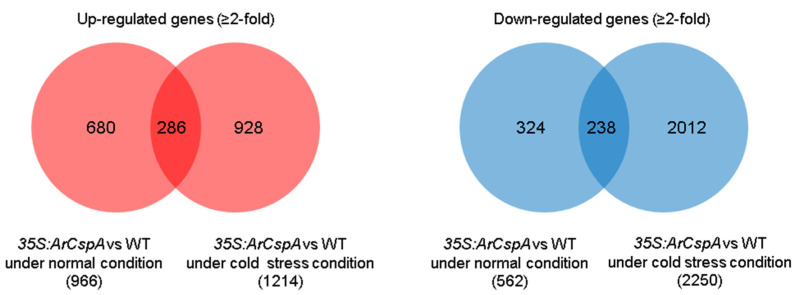
Transcriptome analysis via RNA-seq of the genes involved in the cold-stress response of *35S:ArCspA* transgenic plants. Venn diagrams show the numbers of genes up- and down-regulated under normal and cold-stress conditions compared to those in the WT. Under normal conditions, 966 and 562 genes were significantly up- and downregulated, respectively. *S:ArCspA* transgenic plant (line #2) compared with the WT plants (*p*-value ≤ 0.1 and fold-change ≥2). Under cold-stress conditions, 1214 and 2250 genes were up- and downregulated, respectively, in the *35S:ArCspA* transgenic plant (line #2) compared to the WT plants (*p*-value ≤ 0.1 and fold-change ≥2).

Under normal growth conditions, 966 and 562 genes were up- and down-regulated, respectively, in *35S:ArCspA* transgenic line #2 (B2) compared with the WT (B) by two-fold, with a p-value less than 0.1 (Figure 4). This indicated that the constitutive expression of the *ArCspA* gene influenced the gene expression profile of the transgenic plants even before cold treatment (Figure 4). Under cold-stress conditions, a total of 1214 and 2250 genes were up- and down-regulated, respectively, in the line #2 (A2) of *35S:ArCspA* transgenic rice compared to the WT (A) by two-fold, with a p-value less than 0.1 (Figure 4). Among them, 286 (15.1%) and 118 (9.2%) genes were up- and down-regulated, respectively, under both normal and cold-stress conditions compared with those in the WT (Figure 4).

To understand the biological function of the DEGs in the *35S:ArCspA* transgenic rice compared with the WT rice, Gene Ontology (GO)-based functional enrichment analysis of the abovementioned up- or down-regulated genes was performed using the web-based tool Geneontology (http://geneontology.org/. p < 0.05). In the biological process category, the GO terms “cellular nitrogen compound metabolic process (GO:0034641)”, “detoxification (GO:0098754)”, and “gene expression (GO:0010467)” were enriched among the upregulated genes in the *35S:ArCspA* transgenic rice under normal conditions (Table 1).

In addition, stress-related GO terms such as “response to stress (GO:0006950)”, “response to cold (GO:0009409)”, and “response to heat (GO:0009408)” were significantly enriched among the upregulated genes in the *35S:ArCspA* transgenic rice (Table 1). These results were consistent with the cold-tolerant phenotype of the *35S:ArCspA* transgenic rice. However, GO terms were not enriched in downregulated genes in the *35S:ArCspA* transgenic rice compared with the WT rice under normal conditions. Under cold-stress conditions, “cell cycle (GO:0007049)”, “chloroplast (GO:0009507)”, and “cell wall (GO:0005618)” were enriched among the downregulated genes in the *35S:ArCspA* transgenic rice (Table 1).

**Figure 5:**
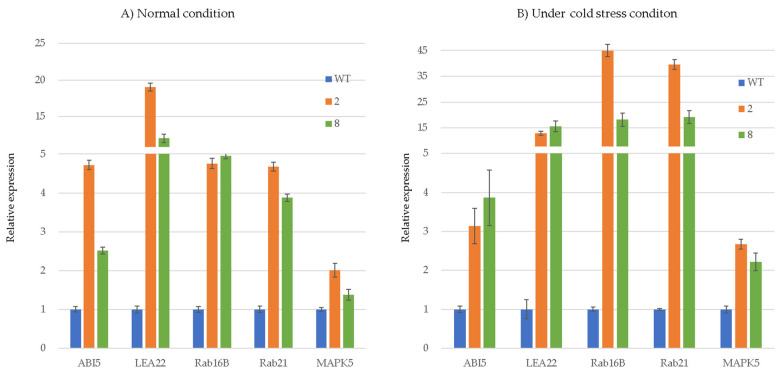
Variation in transcript levels of cold stress-related genes in *35S:ArCspA* transgenic rice and WT plants.

To validate the DEG results, five genes included in the GO term “response to cold (GO:0009409)” were subjected to RT-qPCR with the primer sets (Table S1). Under normal conditions, the expression patterns of five genes, *Rab16B* (Os11g0454200), *Rab21* (Os11g0454300), *LEA22* (Os01g0702500), *ABI5* (Os01g0859300), and *MAPK5* (Os03g0285800), were significantly increased in the *35S:ArCspA* transgenic rice compared to the WT rice (Figure 5A). Five genes (*Rab16B*, *Rab21*, *LEA22*, *ABI5*, and *MAPK5*) were also upregulated in the *35S:ArCspA* transgenic compared with WT rice under cold-stress conditions (Figure 5B).

## 4. Discussion

The ability to cope with low-temperature stress is critical for rice, which originates from subtropical regions. Bacterial CSPs that are highly induced during the cold-shock response are known to be critical for adaptation to cold stress [34]. CSPs are well conserved across all organisms, from bacteria to animals and plants [34]. In an effort to improve the cold resistance of rice, a putative cold-shock protein-encoding gene, *ArCspA*, isolated from *Arthrobacter* cells extracted from soil at the South Pole, was introduced under the control of the *CaMV* 35S promoter (*35S:ArCspA*). To avoid complications due to the location and copy number of the transgenes, 11 transgenic lines were identified as having the insertion within an intergenic region and having a single copy. Phenotypic observations and measurements of chlorophyll contents suggest that these lines performed better against cold stress than the WT and null plants (line #5) did (Figure 2). When *35S:ArCspA* transgenic rice was subjected to cold treatment at 4 °C for 6 days, the recovery rate was much higher than that of the WT (Figure 3). In a previous study, biofunctional analysis using *ArCspA*-overexpressing transgenic *Saccharomyces cerevisiae* showed that *ArCsp* confers cold tolerance to yeast. In addition, transgenic tobacco lines (60.0%) survived after cold treatment at −25 °C for 90 min, whereas under the same treatment conditions, the WT plants did not survive. Taken together, these results clearly indicate that the *ArCspA* protein plays an important role in cold tolerance in higher organisms such as yeast and plants.

RNA-seq analysis was performed on WT and two *35S:ArCspA* transgenic rice line #2 under normal conditions without cold stress and then under cold-stress conditions (Table 1). A total of 966 and 1214 genes were upregulated two-fold and 562 and 2250 genes were downregulated two-fold under normal or cold-stress conditions, respectively, compared with the WT (Figure 4). A comparative differential gene expression (DGE) study was carried out based on these selected genes. Under cold-stress conditions, “cell cycle (GO:0007049)”, “chloroplast (GO:0009507)”, and “cell wall (GO:0005618)” were enriched among the downregulated genes in the *35S:ArCspA* transgenic rice (Table 1). These categories have previously been reported to act either directly or indirectly in abiotic stress [35]. The transcription levels of the genes involved in the photosynthesis, tetrapyrrole synthesis, cell wall, lipid, and nucleotide metabolism are negatively correlated with freezing tolerance, whereas the level of transcription of genes associated with carbohydrate and amino acid and secondary metabolism (e.g., flavonoids) is positively correlated with freezing tolerance [35]. Additionally, stress-related GO terms such as “response to stress (GO:0006950)”, “response to cold (GO:0009409)”, and “response to heat (GO:0009408)” were significantly enriched among the upregulated genes in the *35S:ArCspA* transgenic rice under normal conditions, even without cold-stress treatment (Table 1).

The five genes included in the GO term “response to cold” (GO:0009409) were subjected to RT-qPCR. Our results showed that, compared with the WT, rice overexpressing *ArCspA* presented an increased expression of five genes, *Rab16B*, *Rab21*, *LEA22*, *ABI5*, and *MAPK5*, under both normal and cold-stress conditions. These data suggest that the overexpression of *ArCspA* provided cold tolerance even in the absence of cold-stress treatment by priming the plants to increase the expression of cold stress-related genes, which might function in plant acclimation.

The phytohormone ABA has been shown to be involved in plant developmental processes, abiotic stress tolerance, and senescence. In particular, ABA is especially important in the plant response to environmental stresses such as extreme temperatures (heat, cold, and freezing), drought, and soil salinity [34,36,37]. *Ubi:SeCspA* and *Ubi:SeCspB* transgenic wheat showed improved drought resistance compared with the control plants by stomatal closure following ABA treatment and resulted in an upregulation of *TaCDPK2* and *TaRab18* [23]. Our results showed that ABA-responsive genes (*OsRab21* and *OsRab16B*) were induced in *35S:ArCspA*. *RAB21* has been shown to be induced when plants are subjected to water stress. This gene encodes a basic glycine-rich protein that accumulates in cells upon treatment with NaCl (200 mM) and/or ABA [38]. Compared with *Rab16B* and *Rab16C*, *Rab16A* and *OsRab16D* accumulate at higher levels in ABA-treated germinating seeds [39]. The *Rab16D* expression was not detected in mature seeds, while *Rab16B* and *Rab16C* were only slightly expressed in response to ABA during germination. These findings indicate that *Rab16D* is not a *Lea* gene and suggest that it may lack developmentally regulated promoter element(s) capable of controlling gene expression in seeds.

ABA-dependent signal transcription pathways are highly conserved in rice and Arabidopsis [40,41]. A rice ABA signaling unit composed of *OsPYL*/*RCAR5*, *OsPP2C30*, *SAPK2*, and *OREB1* (*OsABI5*) has been identified to modulate seed germination and early seedling growth [42]. Expression of the *OsABI5* gene was induced after ABA and high-salt treatment, and the mRNA level continuously increased for 24 h. Cold treatment (4 °C) initially suppressed the *OsABI5* expression but then induced it, resulting in a peak at 24 h [43]. *OsABI5* acts as a negative regulator of the salt stress response, such that the overexpression of *OsABI5* in rice provides a high sensitivity to salt stress, while the repression of *OsABI5* promotes stress tolerance and results in low rice fertility. Another bZIP transcription factor, ABI5-Like1 (*ABL1*), is induced in response to the hormones ABA and indole-3-acetic acid, and under stress conditions such as salinity, drought, and osmotic pressure [44]. *ABL1* governs plant stress responses by regulating a series of ABRE-containing *WRKY* family genes to cope with stress tolerance [44].

Mitogen-activated protein kinase (MAPK) signaling is one of the main signal transduction pathways through which environmental signals are conveyed. In a traditional MAPK cascade, signals are transmitted via sequential phosphorylation reactions and activation of MAPK kinase kinase, MAPK kinase (MKK), and MAPK. Calcium (Ca2+) signaling is another critical signaling pathway triggered by environmental stimuli and developmental cues. A rice calcium-dependent protein kinase (CDPK), CPK18, was recently identified as an upstream kinase of MAPK (MPK5), suggesting that MPK5 can modulate these important signal transduction pathways [45]. The response of *MAPK* genes to ABA treatment suggests the involvement of these genes in ABA signaling. *OsMPK3* was induced in rice seedlings in response to a moderately low temperature (12 °C) but not severely low temperature (4 °C). *OsMPK3* is a component of the moderately low-temperature signaling pathway, regulates the cold-stress tolerance of rice and is activated by the upstream OsMKK6 [46].

Genes involved in signal transduction associated with cold tolerance have been targets for developing cold-tolerant plants. In Arabidopsis, the overexpression of transcription factors such as *DREB1*s/*CBF*s, *ZAT12*, and *HSFC1* provides cold tolerance [47,48,49]. Genes involved in the synthesis of compatible solutes, such as proline or raffinose, have been isolated and have been shown to confer both cold and drought tolerance to rice. Specifically, overexpression of the *galactinol synthase DaGolS2* from *Deschampsia antarctica*, which inhabits Antarctica, was shown to be effective in rice. In addition, *OsGolS2* and *GolS* from rice and wheat, respectively, are effective for generating cold-tolerant crops [50]. Interestingly, *OsCAF1B*, which is involved in cytoplasmic mRNA deadenylation, is effective at enhancing rice chilling stress tolerance [51].

How ArCspA confers cold resistance to rice is currently unclear. Rice genes such as *Csp1* (Os02g0121100) and *Csp2* (Os08g0129200) were slightly increased in shoot and root tissues by short-term low-temperature treatment [52]. In contrast, OsCsp proteins accumulate to high amounts in developing panicles, flowers, and seeds, suggesting that genes might have evolved to function in organs that are vulnerable to chilling and freezing stresses. It has been proposed that *CspA* functions as a cold-shock transcriptional activator. The sequence of the CspA protein of *E. coli* is 43% identical to that of the CSD of eukaryotic Y-box proteins, which have an RNA- or DNA-binding ability. CspA has been shown to bind CCAAT box and ATTGG sequences in the promoter regions of hns and gyrA, respectively, while eukaryotic transcription factors bind specifically to inverted CCAAT box sequence elements in gene enhancers and promoters [17,53]. In the RAP database, 32,169 genes were found to have at least one ATTGG or CCAAT sequence within a 1-kb-long promoter region. Moreover, 13,197 genes have more than two elements within their 1-kb-long promoter regions, and among them, the qPCR-verified genes include *LEA22* (Os01t0702500), *ABI5* (Os01t0859300), and *MAPK5* (Os03t0285800). *CspA* may function as an RNA chaperone-like molecule to unwind tightly folded RNA molecules, particularly to prevent the formation of secondary structures within RNA molecules at low temperatures [17]. No specific RNA sequences associated with CspA binding were identified, indicating that CspA has broad sequence specificity. CspA might destabilize RNA secondary structures to make them susceptible to ribonucleases. Such a function may be crucial for the efficient translation of mRNAs at low temperatures and may also affect transcription. Indeed, *CspA* contains the RNA-binding RNP1 sequence motif [G-A]-[FY]-[GA]-[FY]-[IVA] [54].

## 5. Conclusions

Transgenic rice overexpressing a cold-shock protein (*ArCspA*)-encoding gene from the South Pole-dwelling soil bacterium *Arthrobacter* sp. A2-5 present increased cold tolerance. GO analysis suggested that abiotic stress-related terms (GO:0006950, GO:0009409, and GO:0009408) were significantly enriched in *35S:ArCspA* transgenic rice under normal conditions, even without cold-stress treatment. Genes such as *Rab16B* (Os11g0454200), *Rab21* (Os11g0454300), *LEA22* (Os01g0702500), *ABI5* (Os01g0859300), and *MAPK5* (Os03g0285800) were significantly upregulated in these transgenic rice plants compared with the WT rice plants. Taken together, these results indicate that the *ArCspA* gene might be involved in the induction of cold-responsive genes and might provide cold tolerance.

## Figures and Tables

**Table 1:** Biological processes enriched by genes whose expression was upregulated two-fold in *35S:ArCspA* transgenic rice.

Go Function		No. Genes	No. Genes	*p*-Value
		(Reflist)	(Up- or Downregulated)	
Upregulated genes in 35S:ArCspA/WT under normal conditions				
Response to stress	GO:0006950	2115	113	1.47 × 10^−17^
Response to cold	GO:0009409	92	12	2.31 × 10^−6^
Response to heat	GO:0009408	102	13	1.12 × 10^−6^
Response to hormone	GO:0009725	589	31	1.48 × 10^−5^
Cellular nitrogen compound metabolic process	GO:0034641	3075	38	1.36 × 10^−4^
Cellular component organization or biogenesis	GO:0071840	2335	24	3.62 × 10^−5^
Detoxification	GO:0098754	269	16	4.56 × 10^−4^
Gene expression	GO:0010467	1708	16	1.60 × 10^−4^
Upregulated genes in 35S:ArCspA/WT under cold-stress conditions				
Gene expression	GO:0010467	1708	18	2.66 × 10^−6^
Downregulated genes in 35S:ArCspA/WT under cold-stress conditions				
Cell cycle	GO:0007049	410	45	7.46 × 10^−6^
Cell wall	GO:0005618	326	31	1.74 × 10^−3^
Chloroplast	GO:0009507	1414	30	4.68 × 10^−8^

## Data Availability

The raw data are available from the corresponding author upon reasonable request.

## Supplementary Materials

The following are available online at https://www.mdpi.com/article/10.3390/genes12101589/s1, Table S1: Primer sequences. Table S2: Summary of the RNA-seq data. Table S3: The analysis of RNA-Sequencing data in 35S:ArCspA transgenic rice (line #2) compared with WT rice before and after cold-stress treatment.
